# The Termite Fungal Cultivar *Termitomyces* Combines Diverse Enzymes and Oxidative Reactions for Plant Biomass Conversion

**DOI:** 10.1128/mBio.03551-20

**Published:** 2021-06-15

**Authors:** Felix Schalk, Cene Gostinčar, Nina B. Kreuzenbeck, Benjamin H. Conlon, Elisabeth Sommerwerk, Patrick Rabe, Immo Burkhardt, Thomas Krüger, Olaf Kniemeyer, Axel A. Brakhage, Nina Gunde-Cimerman, Z. Wilhelm de Beer, Jeroen S. Dickschat, Michael Poulsen, Christine Beemelmanns

**Affiliations:** a Group of Chemical Biology of Microbe-Host Interactions, Leibniz Institute for Natural Product Research and Infection Biology, Hans Knöll Institute (HKI), Jena, Germany; b Department of Biology, Biotechnical Faculty, University of Ljubljana, Ljubljana, Slovenia; c Lars Bolund Institute of Regenerative Medicine, BGI Qingdao, Qingdao, China; d Section for Ecology and Evolution, Department of Biology, University of Copenhagen, Copenhagen, Denmark; e Kekulé Institute of Organic Chemistry and Biochemistry, University of Bonn, Bonn, Germany; f Department of Molecular and Applied Microbiology, Leibniz Institute for Natural Product Research and Infection Biology, Hans Knöll Institute (HKI), Jena, Germany; g Department of Biochemistry, Genetics and Microbiology, Forestry and Agricultural Biotechnology Institute (FABI), University of Pretoria, Hatfield, Pretoria, South Africa; Max Planck Institute for Marine Microbiology

**Keywords:** symbiosis, lignin degradation, *Termitomyces*, metabolites, redox chemistry, biodegradation, lignocellulose, redox proteins, secondary metabolism

## Abstract

Macrotermitine termites have domesticated fungi in the genus *Termitomyces* as their primary food source using predigested plant biomass. To access the full nutritional value of lignin-enriched plant biomass, the termite-fungus symbiosis requires the depolymerization of this complex phenolic polymer. While most previous work suggests that lignocellulose degradation is accomplished predominantly by the fungal cultivar, our current understanding of the underlying biomolecular mechanisms remains rudimentary. Here, we provide conclusive omics and activity-based evidence that *Termitomyces* employs not only a broad array of carbohydrate-active enzymes (CAZymes) but also a restricted set of oxidizing enzymes (manganese peroxidase, dye decolorization peroxidase, an unspecific peroxygenase, laccases, and aryl-alcohol oxidases) and Fenton chemistry for biomass degradation. We propose for the first time that *Termitomyces* induces hydroquinone-mediated Fenton chemistry (Fe^2+^ + H_2_O_2_ + H^+^ → Fe^3+^ + ^•^OH + H_2_O) using a herein newly described 2-methoxy-1,4-dihydroxybenzene (2-MH_2_Q, compound 19)-based electron shuttle system to complement the enzymatic degradation pathways. This study provides a comprehensive depiction of how efficient biomass degradation by means of this ancient insect’s agricultural symbiosis is accomplished.

## INTRODUCTION

Among the different types of nutritional symbiosis, crop agriculture represents one of the most sophisticated systems. Beyond examples from humans, only a few insect lineages maintain and manure external symbiotic partners (1). Fungus-growing termites (*Macrotermitinae*) underwent a major transition ca. 30 million years ago, when they started to domesticate the mutualistic fungus *Termitomyces* (Agaricales, Lyophyllaceae) as their main food source (2, 3). Since then, fungus-growing termites have become major biomass decomposers of dead plant material, resulting in a substantial ecological footprint in the Old World (sub)tropics (4, 5).

**FIG 1:**
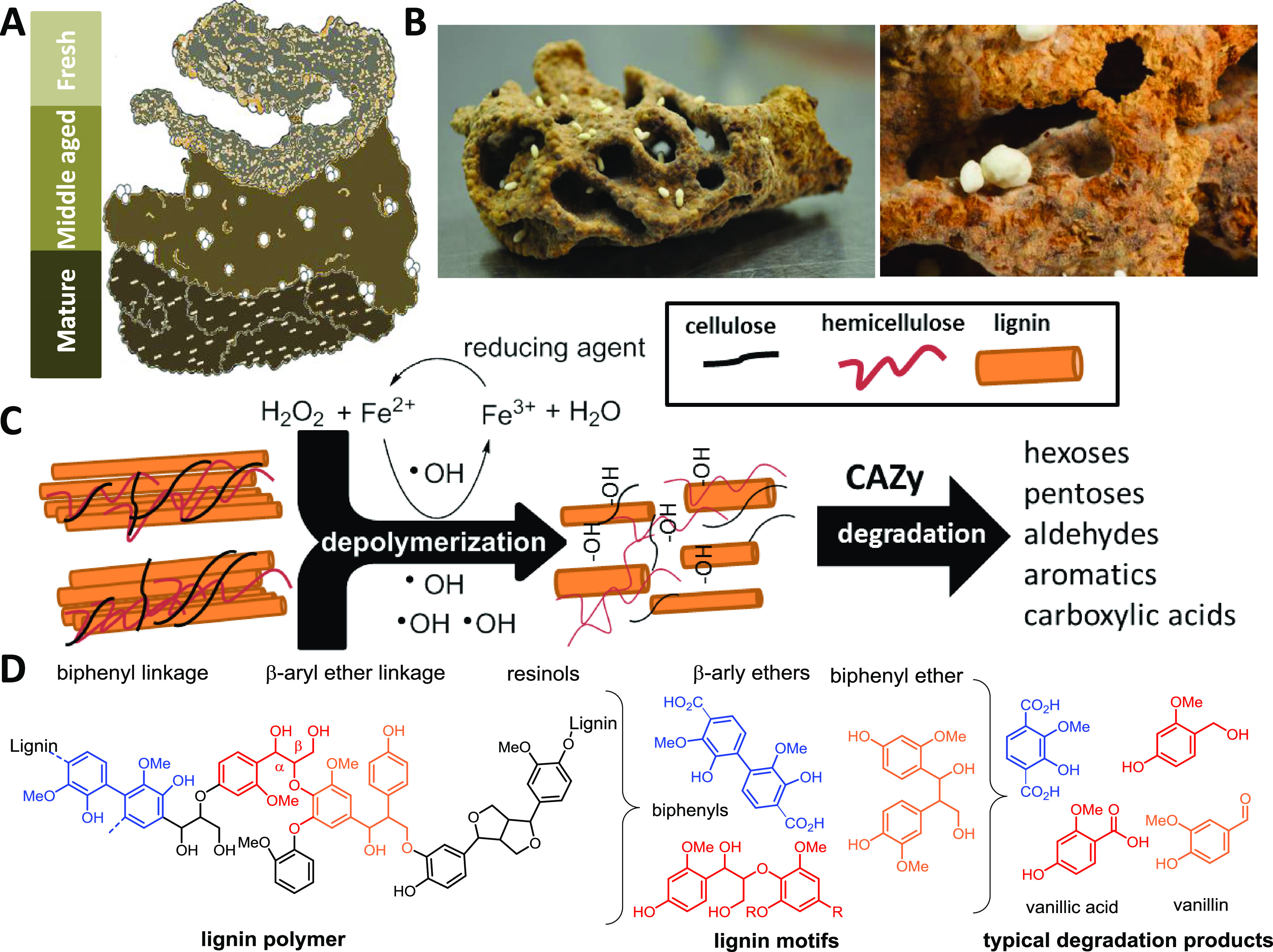
(A) Schematic representation of fungus comb at different maturation stages; (B) freshly collected mature fungus comb carrying fungal nodules; (C) schematic representation of lignin depolymerization via hydroxylation and oxidative cleavage with subsequent degradation by CAZy enzymes to smaller metabolites; (D) schematic structure of lignin biopolymer, lignin motifs, and lignin-derived degradation products.

*Termitomyces* is manured by termite workers in a cork-like structure termed the “fungus comb,” which is found within the underground chambers of the termite mound and is comprised of predigested plant material (Fig. 1A and B) (6). Old termite workers collect and transport a mix of dead plant material (7), while younger workers macerate and ingest the plant material along with asexual *Termitomyces* spores and enzymes, which are produced in fungal nodules on the mature parts of the fungal comb (1, 2). The resulting lignocellulose and spore-enriched feces is then used to craft fresh fungus comb. After spore germination, the fungus matures within 15 to 20 days, and energy-rich fungal nodules are formed to serve as the major food source for younger workers (8). During an average turnover time of 45 to 50 days, the remains of the comb material serve as the major nutrition of older workers, resulting overall in the nearly wasteless decomposition and recycling of plant material (9).

Although the feeding behavior of termites has been studied in detail for decades (10), the underlying biochemical mechanisms for degrading the foraged plant biomass have remained largely unresolved and are the topic of intense discussion (1, 11). Plant biomass consists mostly of lignocellulose, a complex matrix consisting of cell wall polysaccharides: cellulose (40 to 50%), hemicellulose (25 to 30%), and the structurally complex and inhomogeneous phenolic polymer lignin (15 to 20%) (12). The depolymerization and degradation of lignin provides an enormous energetic burden to any microorganism due to lignin’s recalcitrant nature and the strong covalent carbon-carbon and carbon-oxygen linkages between hydroxycinnamoyl alcohol-derived monomers that are covalently cross-linked to plant polysaccharides (Fig. 1C and D) (13, 14). However, once oxidative mechanisms have broken up the dense lignin structure, degrading enzymes are able to diffuse into the material and access the embedded biphenylic, phenolic, and carbohydrate reservoirs for further biomass conversion (15–17).

Although the degradation process appears to be a necessary endeavor to manure the product of the complex fungus-termite-bacterium symbiosis, the fate of lignin within termite fungus combs still remains unclear. A recent study on fungus comb pretreatment in Odontotermes formosanus by Li et al. indicated that lignin is partly cleaved during the first gut passage (18). Additionally, it was hypothesized that *Termitomyces* might have lost key delignification potential throughout its evolutionary history with the termites. However, previous and more recent transcriptomic and analytically guided studies of other *Macrotermitinae* species by da Costa and coworkers showed that fresh comb from *Odontotermes* spp. and Macrotermes natalensis is lignin rich (7), suggesting that the role of gut passage in lignin cleavage may differ between termite species (9). Based on fungal transcriptome sequencing (RNA-seq) analysis and enzymatic assays, the study reasoned that maturation of the fungus comb causes the decomposition of the lignocellulose-rich biomass through the actions of fungal and/or bacterial enzymes.

These partially contradictory results led us to investigate whether *Termitomyces* has the capacity to depolymerize or even degrade lignin-rich biomass. Hence, we commenced our analysis by a comparative genome analysis of nine *Termitomyces* species and an assessment of their capacity to produce ligninolytic enzymes (e.g., laccase [EC 1.10.3.2], lignin peroxidase [LiP; EC 1.11.1.14], manganese peroxidase [MnP], oxygenase [unspecific peroxygenases [UPOs]; EC 1.11.2.1], and versatile peroxidases [VPs] [19] as well as other enzymes supporting degradative pathways and protecting against oxidative stress) (20–22). Here, we show that all investigated *Termitomyces* have similar repertoires of carbohydrate-active enzymes (CAZymes), including a conserved set of ligninolytic enzymes (a MnP, a dye-decolorizing peroxidase [DyP], UPOs, and laccases) with a broad aromatic and phenolic substrate spectrum (23, 24), but lack other class II peroxidases (e.g., LiPs, VPs) that are known to readily oxidize the more recalcitrant nonphenolic moieties of lignin, as described for other basidiomycete white-rot fungi (13, 14, 16). Our findings were supported by the analysis of gene expression levels in RNA-seq data sets obtained from fungus comb at different maturation stages (7). Additional *in silico* and biochemical studies led us to the conjecture that *Termitomyces* might employ hydroquinone-mediated Fenton chemistry (Fe^2+^ + H_2_O_2_ + H^+^ → Fe^3+^ + ^•^OH + H_2_O) using a herein newly described 2-methoxy-1,4-dihydroxybenzene (2-MH_2_Q; compound 19)-based electron shuttle system to complement enzymatic lignin degradation pathways. We further deduced that the presence of small dicarboxylic acids produced by *Termitomyces* not only allows the fungus to solubilize necessary metal ions but also mediates Fenton-based redox chemistry, making the system one of the most effective farming insect symbioses.

## RESULTS

### Genomic and transcriptomic analysis of lignocellulolytic capacity.

First, we subjected two *Termitomyces* species, excavated in South Africa in 2011 and 2015, to whole-genome sequencing using Illumina sequencing technology (LGC Genomics [Berlin, Germany]) and RNA sequencing using the BGISeq-500 platform (BGI, Hong Kong). Annotated genomes of both species were obtained using AUGUSTUS 3.3.3 after RNA-seq data were mapped to the genomes and used for algorithm training. The resulting draft genome of *Termitomyces* sp. strain T153 (Macrotermes natalensis) had an estimated size of 84.1 Mb (scaffold *N*_50_ = 23.88 kb), with more than 13,000 genes (GenBank accession no. JACKQL000000000). Similarly, the draft genome of *Termitomyces* sp. strain T112 (*Macrotermes natalensis*) had an estimated size of 79.8 Mb (scaffold *N*_50_ = 33.34 kb) and also >13,000 genes (accession no. JACKQM000000000). For further analysis, we also reannotated seven *Termitomyces* genomes deposited in GenBank, including our previously reported *Termitomyces* sp. strain J132 (alias P5) from *Macrotermes natalensis* (3), using the same settings in AUGUSTUS 3.3.3 (Tables S2 and S3 at https://doi.org/10.5281/zenodo.4431413). To gain insights into the functional capacity for biomass degradation, we first identified CAZyme families within each genome using a local installation of the dbCAN2 server (25–27).

**FIG 2:**
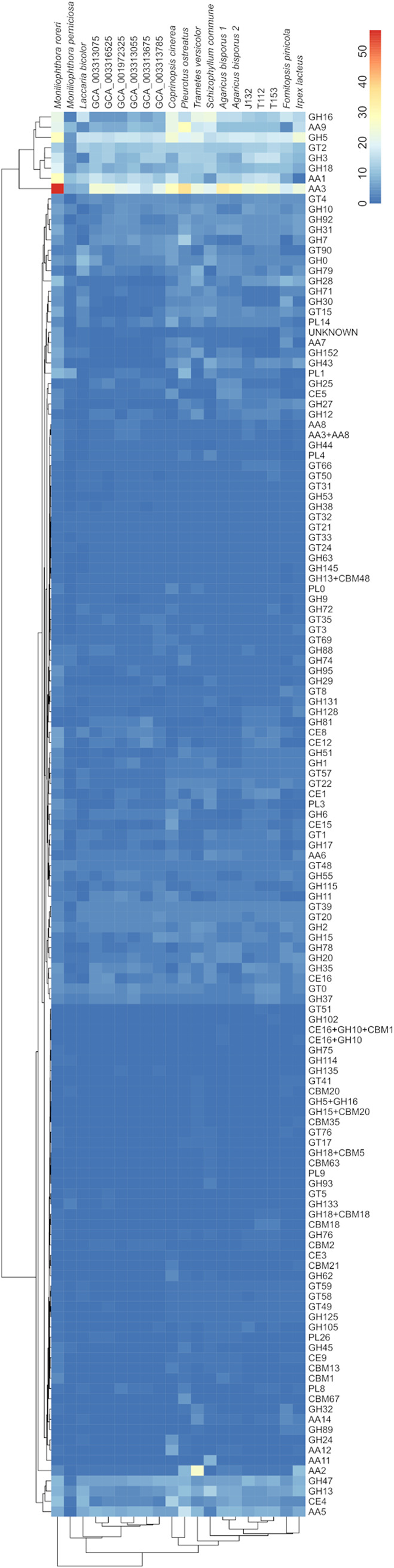
Heatmap of the numbers of hits for representatives of different CAZy families in the predicted proteomes of *Termitomyces* spp. (T112, T153, J132, GCA_001972325, GCA_003313055, GCA_003313075, GCA_003313675, GCA_003313785, GCA_003316525) and other selected basidiomycete fungi (Laccaria bicolor, Moniliophthora perniciosa, Moniliophthora roreri, Agaricus bisporus var. *Burnettii* [Agaricus bisporus 1], *Agaricus bisporus* var. *Bisporus* [Agaricus bisporus 2], Coprinopsis cinerea, Schizophyllum commune, Fomitopsis pinicola, Trametes versicolor, Pleurotus ostreatus, Irpex lacteus). The vertical axis shows clustering of enzymes based on abundance.

Comparison of all nine *Termitomyces* genomes revealed that all species had relatively similar predicted proteomes with comparable numbers of polysaccharide-degrading enzymes, such as exo-cellobiohydrolases, endoglucanases assigned to different glycoside hydrolase (GH) families, and lytic polysaccharide monooxygenase (LPMOs), but no particular enrichment or reduction of CAZy families compared to those of other basidiomycete reference genomes was found (Fig. 2; see Fig. S1 and S2 at https://doi.org/10.5281/zenodo.4431413) (28).

We then specifically searched *Termitomyces* genomes for the presence/absence of gene sequences encoding highly oxidizing enzymes that could contribute to the depolymerization and catabolic degradation of lignin (Fig. 2; see Table S4 at https://doi.org/10.5281/zenodo.4431413) (18). It is worth noting that *Termitomyces* genomes contained, on average, 16 gene sequences encoding laccases (AA1) (29–32), oxidases with low redox potential that use diphenols and related substances as electron donors and oxygen as the acceptor, thereby creating reactive C and O-based radical species in the process. In addition, we identified one putative MnP (AA2) per genome, an enzyme that generates highly reactive Mn^3+^ species that, once chelated, are able to diffuse through the dense network of lignocellulose, causing oxidation degradation due to their higher redox potential (23). Furthermore, a subset of gene sequences encoding alcohol oxidases and dehydrogenases (AA3 and AA5) are known to catalyze the oxidation of (aryl)-alcohols or carbohydrates with the concomitant formation of hydroquinones and/or H_2_O_2_ (33, 34). Unlike in previous studies (1), we also identified sequences encoding a DyP and an UPO (Tables S5 and S6 at https://doi.org/10.5281/zenodo.4431413), both of which are known for their versatile substrate spectrum. However, homologous sequences to other class II peroxidases (PODs), such as LiPs, were not detectable. Along these lines, iron reductase domains (AA8, EC 1.16.1) and putative benzoquinone reductases (AA6, EC 1.6.5.6) that are key to maintaining efficient Fenton chemistry-based redox cycles by reductive Fe^2+^ sequestration and regeneration of organic benzoquinone-based redox shuttles were identified.

**FIG 3:**
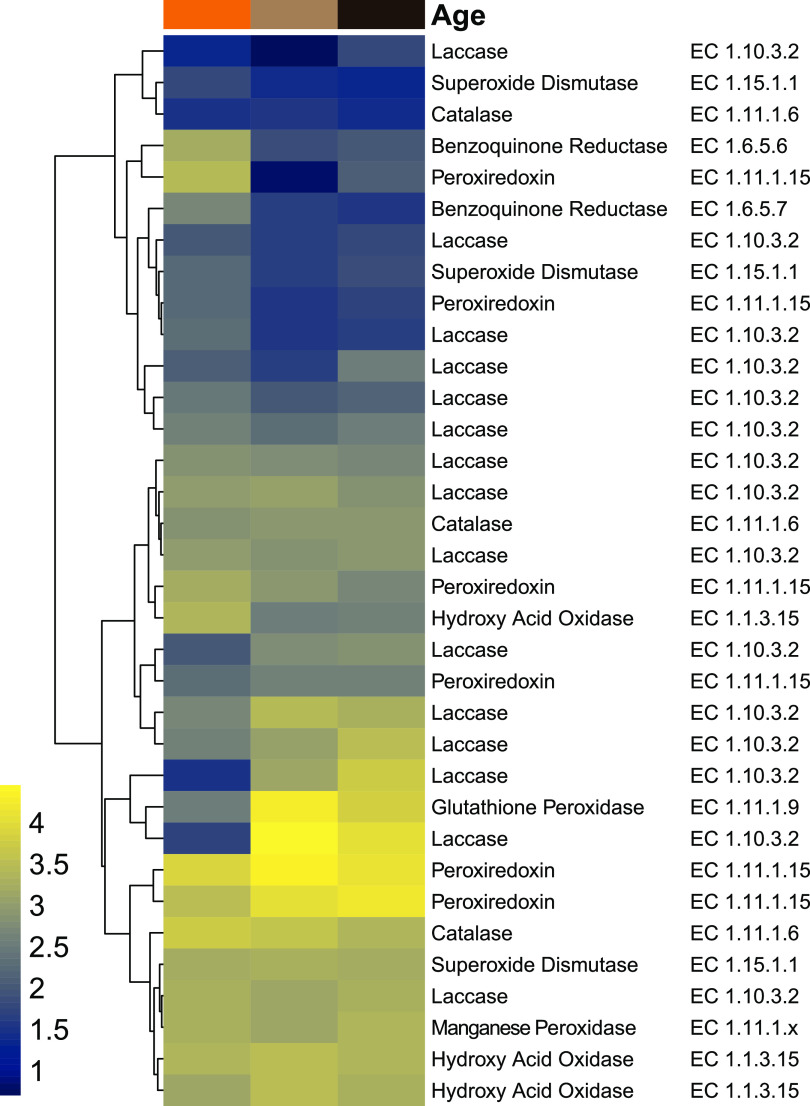
Heatmap of redox enzyme transcription levels based on RNA-seq data of fresh comb (light brown), old comb (black) and nodules (orange) from *Macrotermes* colony Mn156 (7). Transcript abundances are depicted as log_10_ gene expression values, and color schemes were generated by viridis (version 0.5.1) (68, 71).

Subsequently, the expression levels of candidate genes related to lignin depolymerization were analyzed in RNA-seq data obtained from three regions in the fungus comb (Fig. 3) (7): fresh comb (within which most plant biomass decomposition is likely to occur), old comb (where decomposition might still occur but to a lesser extent), and nodules as (which feed young workers and serve for fungal spore and enzyme transport) (Fig. 3; Table S27 at https://doi.org/10.5281/zenodo.4431413).

Here, we found differentiating transcription levels of genes encoding oxidative enzymes (such as laccases, a MnP, and a UPO) and enzymes of the CAZy families AA3 and AA5, as well as enzymes that protect against reactive intermediates (e.g., benzoquinone reductase [EC 1.6.5.7], superoxide dismutase [EC 1.15.1.1], glutathione peroxidase [EC1.11.1.9], and peroxiredoxin [EC 1.11.1.15]) across all three data sets. The combined genetic and transcriptomic survey revealed that *Termitomyces* has the capacity to produce lignocellulolytic enzymes and may even be able to induce and catalyze Fenton chemistry (35).

### Fenton chemistry of *Termitomyces*.

Fenton chemistry involves the reaction between Fe^2+^ and H_2_O_2_, yielding Fe^3+^ and a highly reactive hydroxyl radical (^•^OH), a powerful oxidant (E_0_ = 2.8 V versus that of a normal hydrogen electrode) that is able to unselectively oxidize hydrocarbons and nonphenolic aromatic units within lignocellulose-rich material. Brown-rot fungi are known to make use of Fenton chemistry to depolymerize lignocellulose biomass (36) and modulate the redox potential of Fe^2+/3+^ species by secretion of dicarboxylic acids that act as chelators to form diffusible Fe complexes and as proton donors for catalytic degradation processes (37). Additionally, redox-active fungal quinones (Q) and hydroxyquinones (H_2_Q), such as 2,5-dimethoxy-1,4-benzoquinone (2,5-DMQ), 2,5-dimethoxy-1,4-hydroquinone (2,5-DMH_2_Q), and its regioisomer 4,5-dimethoxy-1,2-benzendiol (4,5-DMH_2_Q), have been discussed to serve as redox shuttles (3 H_2_Q + 2 O_2_ → 3 Q + 2 H_2_O + 2 HO^•^) in the Fenton chemistry of rotting fungi (e.g., Serpula lacrymans, the *Gloeophyllales*, and the *Polyporales*) (38–40), as they have the ability to switch between oxidation states via one-electron transfer reactions that allow for the concomitant formation of Fe^2+^ from Fe^3+^ and hydroxyl radicals (HO^•^) from H_2_O_2_ and O_2_ (see Fig. 5 and 6).

**FIG 4:**
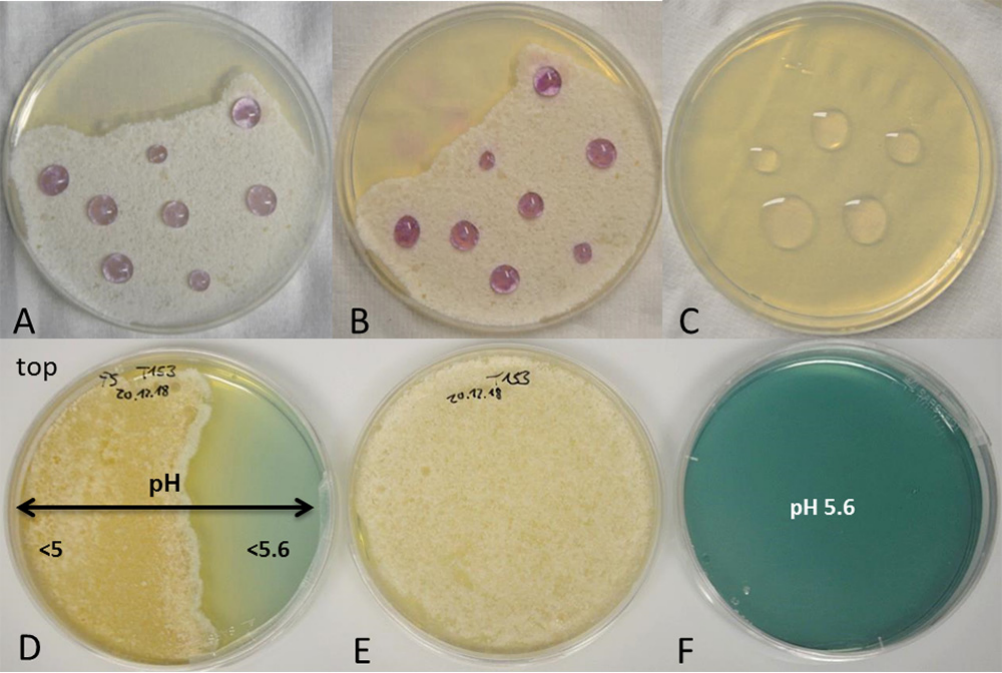
(A and B) Ferrozine solution added to a *Termitomyces* sp. T153 culture grown on PDA (18 days) and incubated for 5 min (A) and 30 min (B); (C) ferrozine solution on a PDA plate (negative control); (D and E) *Termitomyces* sp. T153 grown on PDA (28 days) containing bromocresol green as a pH indicator (D) and without the indicator (E); (F) PDA plate containing bromocresol green (negative control).

Thus, we evaluated whether *Termitomyces* employs any of those measures to enable lignin depolymerization by using *Termitomyces* sp. T153 and P5 as model strains. First, we employed a standardized colorimetric ferrozine assay to determine if extracellular Fe^3+^ is reduced to Fe^2+^ within the surrounding mycelium, a prerequisite to initiate Fenton chemistry (41, 42). As depicted in Fig. 4A, topical application of a ferrozine solution caused a clear color change within minutes, which was indicative of the immediate reduction of Fe^3+^ to Fe^2+^. Next, we determined the pH range within the fungal mycelium, as enzyme activities, the redox potential of H_2_O_2_, and metal complexes are strongly pH dependent (35). We found indications that *Termitomyces* acidifies the surrounding medium (Fig. 4D), which would benefit enzyme activities of lignin-degrading enzymes with a pH optimum of 4.5 to 5.0 (14, 21). As the Fenton reaction also requires H_2_O_2_, we tested if *Termitomyces* generates sufficient extracellular H_2_O_2_ to initiate the reaction. Based on an H_2_O_2_-dependent colorimetric assay, we found that *Termitomyces* generates approximately 4 to 6 μg extracellular H_2_O_2_ per gram mycelium during growth on solid support (mycelium age, 7 to 21 days) (Tables S18 to S20 at https://doi.org/10.5281/zenodo.4431413).

**FIG 5:**
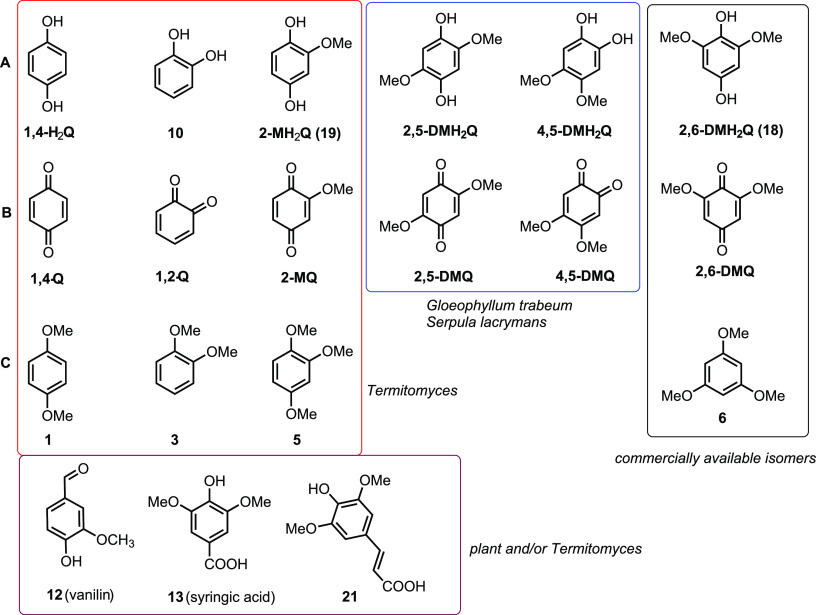
Structures of redox-active compounds discussed in this work. (A) Hydroxyquinones (H_2_Q); (B) corresponding quinones (Q); (C) methoxylated derivatives of H_2_Q. Compounds identified from *Termitomyces* are highlighted in a red box, compounds identified from other rotting fungi are marked with a blue box, derivatives isolated from *Termitomyces* and of plant origin are highlighted in a purple box, and commercial derivatives for comparison are highlighted in a black box.

In a next step, we evaluated if *Termitomyces* produces redox-active H_2_Q/Q using gas chromatography coupled with mass spectrometry (GC-MS). Although the formation of previously reported 2,5-DM(H_2_)Q was not observed, we were intrigued to detect 2-methoxy-1,4-benzoquinone (2-MQ), its reduced H_2_Q named 2-methoxy-1,4-dihydroxybenzene (2-MH_2_Q), and the fully methylated derivative 1,2,4-trimethoxybenzene (compound **5**), as well as other structurally related (di)methoxylated hydroxybenzenes (e.g., compounds **1, 3**, and **12**) (Fig. 5). Additionally, we verified the identities of the newly detected quinone derivatives 2-MQ and 2-MH_2_Q by synthesis and comparison of GC-MS retention times (Fig. S20 and S21 and Tables S16, S17, and S24 at https://doi.org/10.5281/zenodo.4431413).

**FIG 6:**
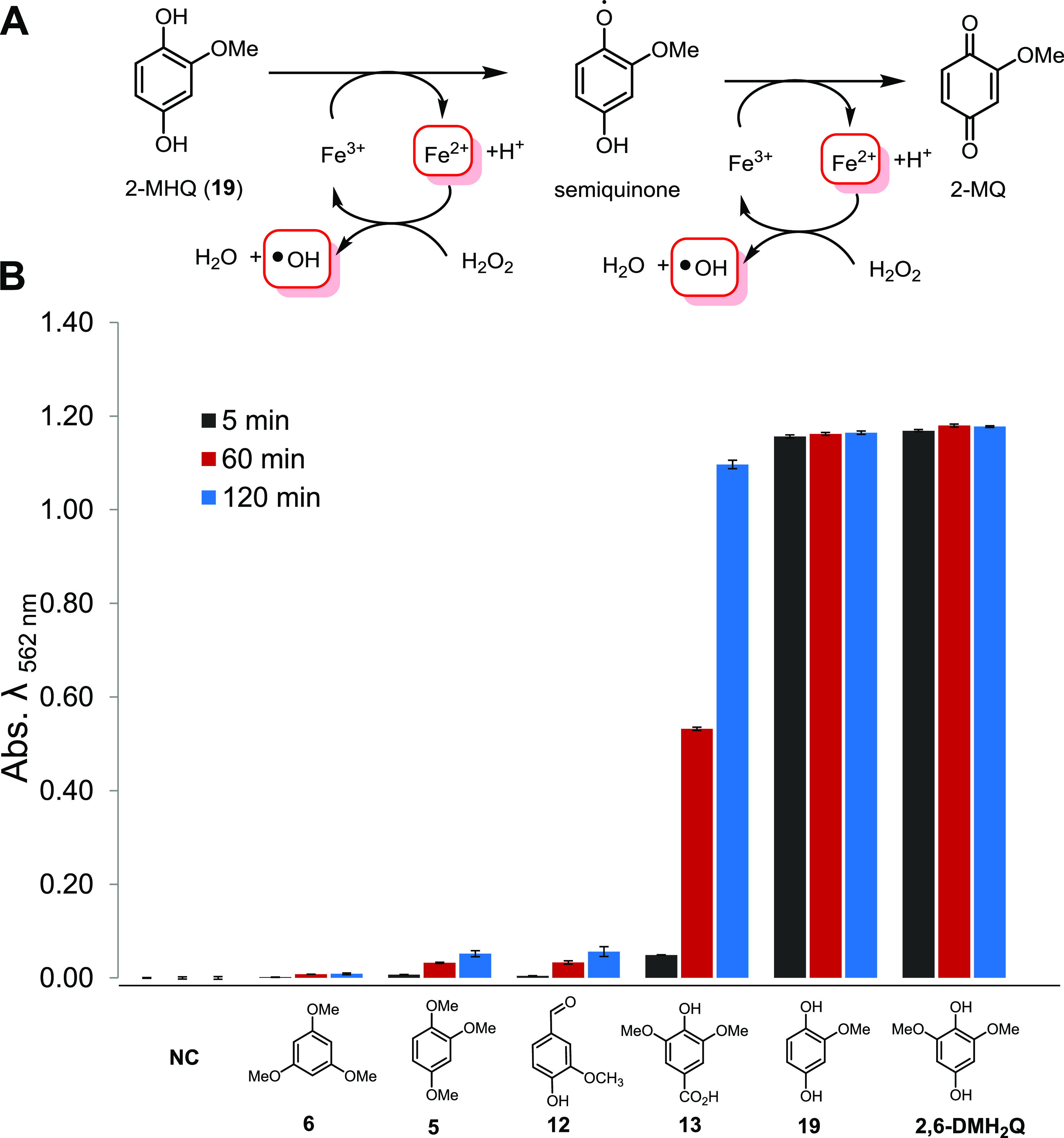
(A) Mechanistic depiction of the 2-MH_2_Q-initiated Fenton reaction via the formation of a radical semiquinone species and oxidation to 2-MQ; (B) quantification of Fe^3+^ reduction by H_2_Q using a colorimetric ferrozine-based assay (sodium acetate [NH_4_OAc] buffer, pH 4). Error bars indicate ±0.5 standard deviation (*n* = 3). Abs. λ_562 nm_, absorbance at a wavelength of 562 nm; NC, negative control.

To evaluate the ability of H_2_Qs to reduce Fe^3+^ to Fe^2+^, we employed the established ferrozine-based Fe^3+^ reduction assay (43). Overall, 2,6-DMH_2_Q (compound **18**), a regioisomer of 2,5-DMH_2_Q, was the most reactive derivative that was able to reduce Fe^3+^ to Fe^2+^ within seconds and was therefore used as positive control in further experiments (Fig. 6). In comparison, 2-MH_2_Q (compound **19**) showed a reduced reactivity, which is likely a reflection of the decreasing electron density of the aromatic system due to the lack of one additional electron-donating –OCH_3_ group. Other tested (methoxylated) hydroxybenzenes showed a reduced reactivity compared to those of compounds **18** and **19**. Subsequently, we expanded our studies to combinations of redox-active derivatives and were able to observe in most cases the superposition of redox activities but no indications of synergistic activity (Fig. S9 and Table S21 at https://doi.org/10.5281/zenodo.4431413).

**FIG 7:**
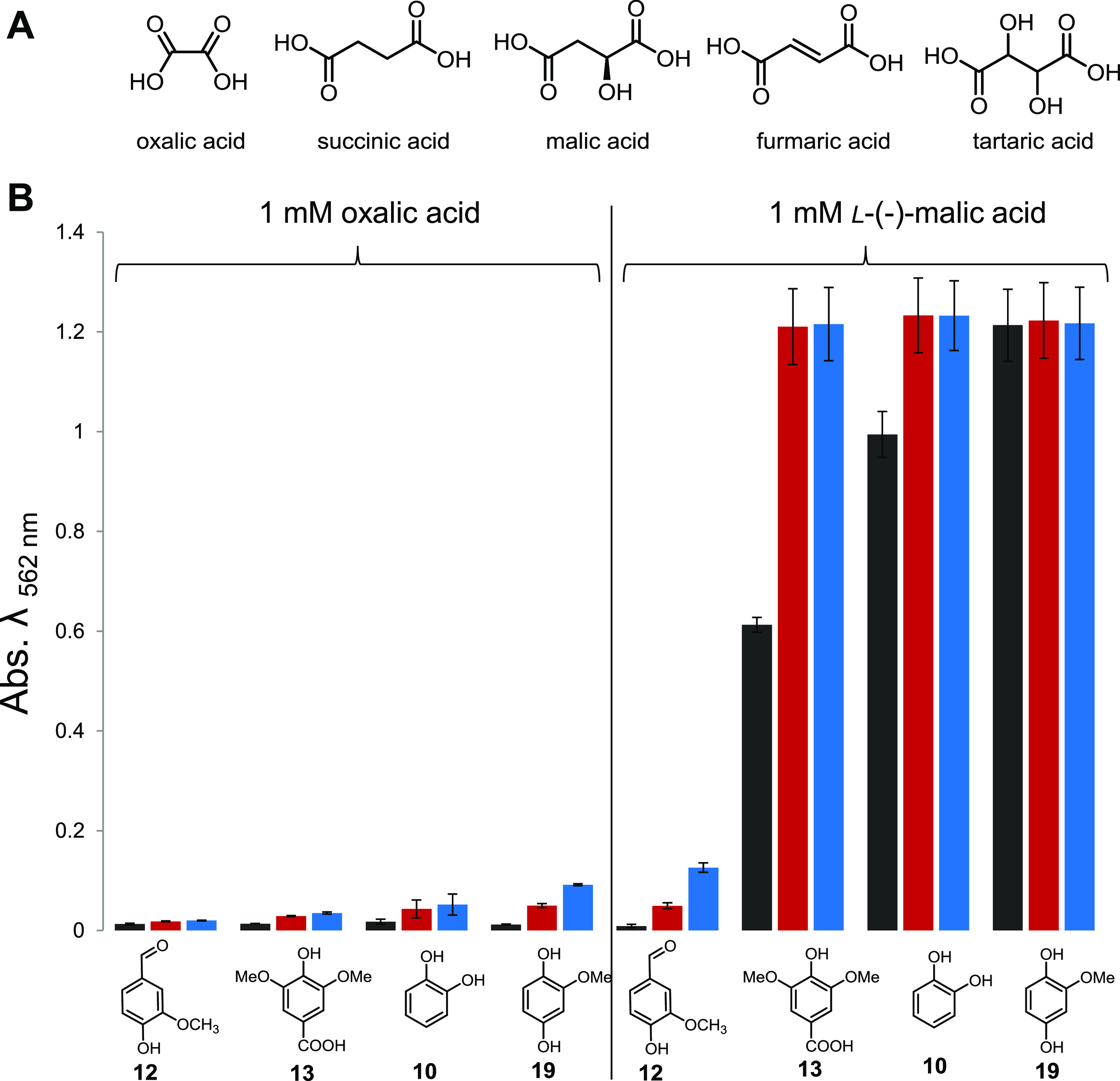
(A) Structures of metal-chelating dicarboxylic acids; (B) quantification of Fe^3+^ reduction by H_2_Q using a colorimetric ferrozine-based assay in in the presence of 1 mM oxalic acid and 1 mM L-(-)-malic acid after 5 min (black), 60 min (red) and 120 min (blue). Error bars indicate ±0.5 standard deviation (*n* = 3).

As Fenton chemistry produces highly reactive hydroxyl radicals (^•^OH), we then confirmed the presence of these short-lived radicals in our H_2_Q-mediated Fenton reactions using a fluorometric assay based on the reaction with terephthalic acid (TPA). As in literature reports for 2,6-DMH_2_Q (compound **18**) (37–40), the newly identified and structurally related H_2_Q compound **19** catalyzed the formation of ^•^OH in the presence of H_2_O_2_ and Fe^3+^ within seconds. In contrast, derivatives such as 1,2-dihydroxybenzene (compound **10**) and syringic acid (compound **13**) caused the formation of hydroxyl radicals with lower initial reactivities, but they formed over a period of more than 90 min (Fig. S5 at https://doi.org/10.5281/zenodo.4431413). Having verified that *Termitomyces* produces reactive H_2_Qs that are able to induce the formation of Fenton reagents (Fe^2+^, H_2_O_2_, and ^•^OH), we then elaborated on the influence of fungus-derived dicarboxylic acids (oxalic acid, tartaric acid, malic acid, fumaric acid, and succinic acid) (44–46) on the Fenton reaction (Fig. 7). While at low concentrations of oxalic acid (0.1 mM) most H_2_Qs were still able to reduce the formed Fe^3+^ complexes, increasing concentrations caused the formation of stable Fe complexes with altered redox potentials, such that only the most reactive, 2,6-DMH_2_Q (compound **18**), was able to reduce Fe^3+^ to Fe^2+^ (Fig. S10 to S12 at https://doi.org/10.5281/zenodo.4431413) (46). At 10 mM oxalic acid, a significant amount of autoxidation-related Fe^3+^ reduction was observed. A similar reactivity trend, albeit with a stronger autoxidation effect, was observed when tartaric acid was investigated as a chelating agent (47). In contrast, the presence of malic, fumaric, or succinic acid only moderately altered the redox potential of the Fe-complexes, and only low rates of autoxidation were observed (Fig. S14 to S16 at https://doi.org/10.5281/zenodo.4431413).

While laboratory culture conditions generally supply sufficient Fe concentrations for growth, we questioned whether or not the natural fungal comb environment provides the necessary metal ions for Fenton chemistry (48). To answer this question, we analyzed the element composition of fungus comb, gut fluids of termite workers, and soil samples derived from within and outside termite colonies from different locations using inductively coupled plasma atomic emission spectrometry (ICP-AES) (49). All tested samples contained Al, Fe, and Ti as some of the most abundant main elements, in addition to significant amounts of Mn. However, amounts of elements important for growth (C, H, P, K, Ca, Mg) were low in all soil samples, with a particularly strong depletion of phosphorus, but potassium was enriched compared to levels in comb and gut samples (Fig. S22 to S29 and Tables S15, S25, and S26 at https://doi.org/10.5281/zenodo.4431413). Sequential ion extraction of soil samples was performed to analyze the soluble metal ion content, and only low concentrations of most metal ions were detectable (50, 51). Although these findings indicate that fungus comb and the gut environment accommodate large amounts of insoluble Fe/Al oxides, the nano- and microscopic surface areas of these minerals may act as the necessary catalytic centers for Fenton-like redox chemistry (52).

### Enzyme activity tests catalyzing the degradation of model lignin compounds.

We then questioned if enzymatic degradation of lignin or lignin-like model substances by *Termitomyces* is measurable using colorimetric assays or MS-based analytical tools (53). For a first test, we supplemented the culture medium of *Termitomyces* sp. T153 with the pigment-based model substance Azure B (54), previously used to measure the redox activity of LPs due to its stability toward oxidative activities of MnPs. Over a time course of seven days, we were able to monitor the decolorization of Azure B by *Termitomyces*, an effect which became more pronounced with the increasing biomass and age of the fungus culture (Fig. S16 at https://doi.org/10.5281/zenodo.4431413). To evaluate if the degrading activity was due to the activity of secreted oxidative enzymes and/or H_2_Q-mediated Fenton-based chemistry, we tested both effectors separately and in combination. While quantification of enzymatic effects was hampered by technical challenges due to intrinsic light absorption of enzymes concentrates, H_2_Q-mediated Fenton chemistry clearly induced the degradation of Azure B within 5 to 10 min in a comparison with the control (Fenton reagents without H_2_Qs) (Fig. S16 at https://doi.org/10.5281/zenodo.4431413) (55). We then evaluated whether or not laccase activity was detectable within the secretome using a syringaldazine-based assay and compared the activity to the reactivity of a commercial laccase from *Trametes versicolor* (56). However, only residual laccase activity was detectable compared to the activity in the positive control and thus was unlikely accountable for the degradation of Azure B.

**FIG 8:**
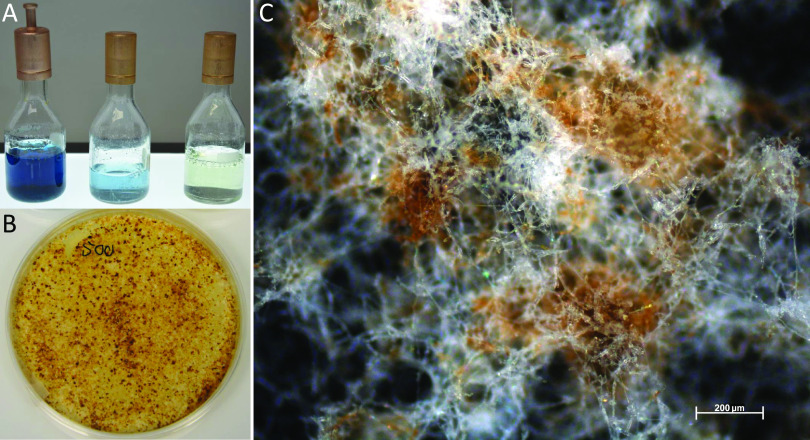
(A) PDB containing Leukoberbelin blue (left to right, culture of *Termitomyces* sp. 153, cell-free supernatant, and PDB broth as a control); (B) *Termitomyces* sp. T153 cultivated on PDA containing 500 μM MnCl_2_ after 28 days; (C) microscopic image of fungal mycelium after 24 days showing brown MnO_2_ deposits.

Lastly, we evaluated if *Termitomyces* exhibits MnP enzymatic activity, which is marked by the oxidation of Mn^2+^ to Mn^3+^ and the release of the highly reactive oxidant as a carboxylic acid chelate, using a previously reported leukoberbelin blue test (57). As shown in Fig. 8, leukoberbelin-containing *Termitomyces* cultures and cell-free culture supernatant resulted in the formation of the blue leukoberbelin complex within minutes, which indicated the formation of Mn^3+/4+^ species. When *Termitomyces* was grown on potato dextrose agar (PDA) plates containing both elevated Mn^2+^ concentrations (200 to 500 μM) and indicator dye, the formation of blue leukoberbelin-Mn^3+/4+^ complexes was detectable within a few days, and longer incubation times resulted in macroscopic MnO_x_ precipitates forming around fungal hyphae within 10 to 17 days (Fig. 4C). We further confirmed the expression of the gene encoding the putative MnP by reverse transcription-PCR (RT-PCR) (Fig. S18 and S19 at https://doi.org/10.5281/zenodo.4431413).

### Proteomic analysis.

Building on our enzymatic studies and to link the observed activities with their putative enzymatic origins, we conducted a liquid chromatography tandem mass spectrometry (LC-MS/MS)-based proteomic analysis of secreted enzymes of *Termitomyces* culture supernatants, which were prepared in two different buffer systems (NaOAc, pH 4.5; KH_2_PO_4_, pH 6.5). Overall, a total of 255/303 secreted proteins were detectable, which were mostly assigned to fungal carbohydrate metabolism groups, such as glucosidases, glucanases, or chitinases (Tables S29 to S32 at https://doi.org/10.5281/zenodo.4431413). Interestingly, a potential lignin-degrading aromatic peroxygenase (8^th^/13^th^) and one MnP (13^th^/11^th^) ranked among the top 15 most abundant protein sequences, while two other yet-unassigned peroxidases were also detectable (31^st^, 141^th^/17^th^, 142^nd^), albeit at lower abundances. In total, five laccases were detectable, albeit in minor abundances (starting from 76^th^/99^th^).

## DISCUSSION

In the two major fungus-growing termite genera, *Macrotermes* and *Odontotermes*, the decomposition of plant biomass by the fungal cultivar *Termitomyces* is based on the intricate interactions between the predigestive gut passage and the external fungus comb bioreactor. Although a series of studies have elaborated on the functional roles of *Termitomyces* in plant biomass degradation (1–3), experimental insights into the biochemical mechanisms necessary for plant biomass degradation have remained sparse.

### Which ligninolytic enzymes are produced by *Termitomyces*?

**FIG 9:**
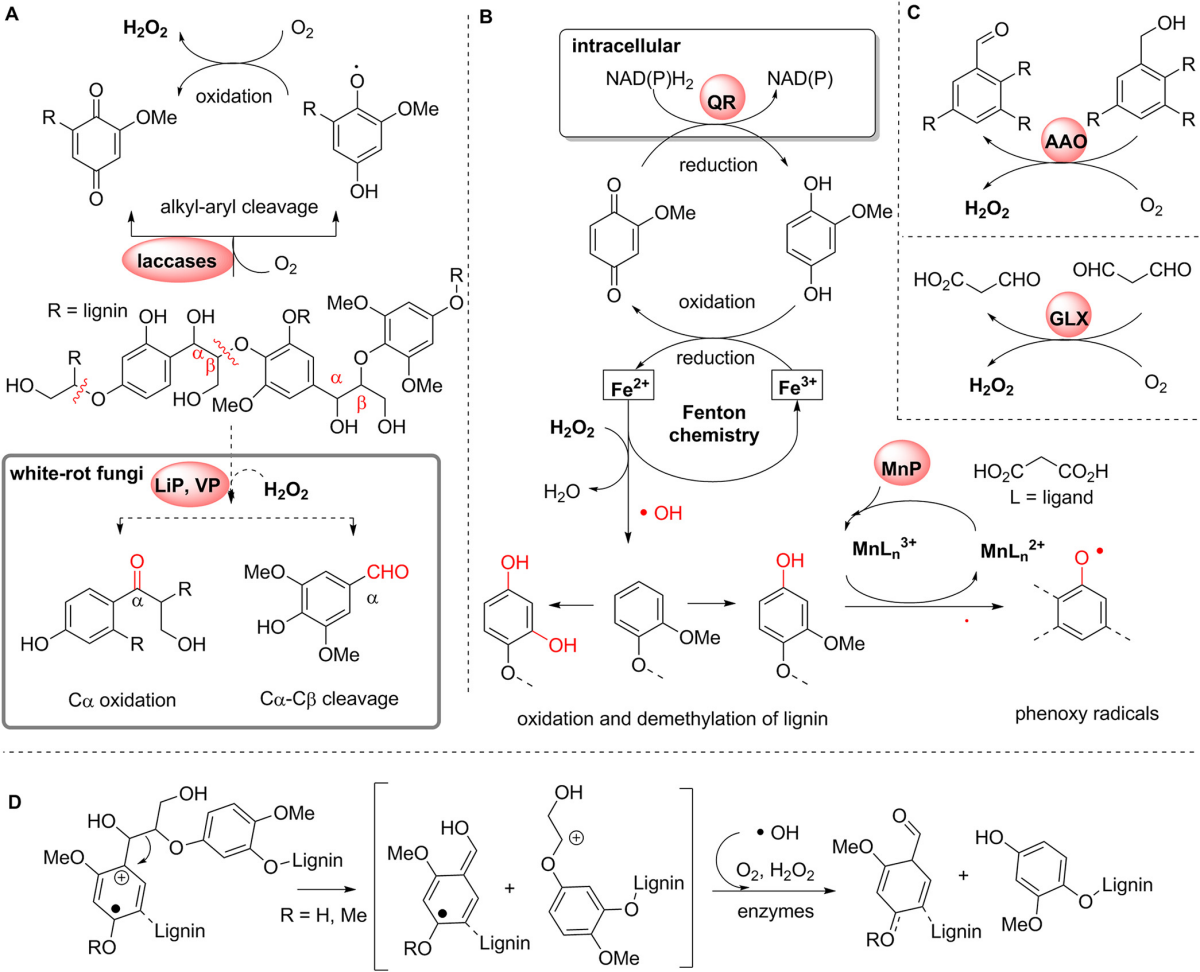
Lignin modifications and oxidation pathways by *Termitomyces*. (A) Schematic depiction of lignin oxidation by, e.g., laccases (Lac) in contrast to degradation by LiP and VP typically found in white-rot fungi (gray box); (B) oxidation and oxidative demethylation of lignin substructures by 2-MH_2_Q-catalyzed Fenton chemistry via the formation of short-lived hydroxyl radicals and regeneration of H_2_Q by (intracellular) benzoquinone reductases (QR); (C) formation of H_2_O_2_ by (aryl)-alcohol oxidases (AAO) and glyoxal oxidases (GLX); (D) oxidative C-C cleavage of lignin substructures via phenoxy and methoxy radicals derived from radicals and/or enzymatic processes.

Our omics-based analysis clearly shows that *Termitomyces* has the capability to produce a set of extracellular lignocellulose‐degrading enzymes, most of which generate diffusible extracellular oxidants (superoxide O_2_^−^, hydroxyl radicals ^•^OH, H_2_O_2_, redox-active Mn^3+/4+^ species, or phenoxy radicals) that oxidize the aromatic polymeric three-dimensional (3D) structure of lignin (Fig. 1D and Fig. 9).

It is particularly intriguing that *Termitomyces* encodes, on average, 16 different laccases that are differentially transcribed and might differ in their reactivities and substrate spectra. Although laccases are considered not to be essential for lignin degradation (22, 23), their presence likely assists in partial oxidation of phenolic and nonphenolic aromatic moieties that facilitate further fragmentation and depolymerization (Fig. 9). Here, it is also worth highlighting that produced (aryl)-alcohol oxidases are able to efficiently oxidize and cleave β-ether units present within lignin substructures via single-electron transfer reactions (22, 23). Our study also provides conclusive genomic and biochemical evidence that *Termitomyces* secretes not only a reactive MnP, a class II peroxidase, but also a DyP and a UPO, both of which are known for oxidizing a broad-substrate spectrum. While none of these enzymes are capable of degrading lignin alone, their combined enzymatic actions should allow for lignin’s partial depolymerization, which is necessary for other enzymes of microbial or termite origin to overcome physical barriers of the polymer and access their target substrates in the interior of the dense polymer.

### Does Fenton chemistry play a role?

Following up on the idea that *Termitomyces* utilizes complementary Fenton chemistry for breaking chemical bonds in the dense lignocellulose, we evaluated the presence and absence of metabolic and enzymatic factors necessary to drive the radical process. Here, we provide collective evidence that *Termitomyces* employs Fenton chemistry by the secretion of high levels of (extracellular) H_2_O_2_ and the production of H_2_Qs that reduce Fe^3+^ to Fe^2+^. For the first time, we document that the *Termitomyces*-derived metabolite 2-MH_2_Q (compound **19**) acts as a redox shuttle for Fenton chemistry and induces the formation of Fe^2+^, as with 4,5-DMH_2_Q and 2,5-DMH_2_Q (21, 35). Genomic and transcriptomic analyses also showed that *Termitomyces* produces two benzoquinone reductases that may reduce MQ to MH_2_Q and thereby close the H_2_Q/Qbased redox shuttle cycle. Considering that Fenton chemistry produces several strong oxidants, we evaluated the influence of fungal dicarboxylic acids on the H_2_Q-based reduction of Fe^3+^ complexes and found that complexation with oxalic acid renders the metal ion less available for reduction in a concentration-dependent manner. Thus, we hypothesize that *Termitomyces* actively applies these protective measures in the proximity of its fungal hyphae, which at the same time allows the sequestering of Fe^3+^ for intracellular processes.

Considering the observation that fungus comb material is crafted from macerated plant material and is interspersed with metal oxide-rich soil, it appears likely that Fenton-based degradation pathways play a major role in the overall biomass conversion during fungal maturation. The importance of Fenton chemistry in plant degradation was recently demonstrated by Schiøtt and Boomsma (58), who showcased that the combination of enzymatic degradation and Fenton chemistry plays an important role in the coevolved leafcutter ant symbiosis, where ants create spatially isolated substrate pellets, called Fenton pellets, that might function as small contained bioconversion reactors.

### Conclusions.

Collectively, our genomic, transcriptomic, metabolomic, and proteomic studies document that *Termitomyces* utilizes a specific set of oxidative enzymes as well as Fenton chemistry to cope with the challenging task of degrading the lignin-rich plant biomass and presumably applies the same mechanism to detoxify xenobiotic compounds present within the comb, such as plant and microbial natural products, which often have structures similar to those of lignin monomers (48, 50, 52). Our findings increase our general understanding of the role of Fenton chemistry within the symbiosis of termites and their cultivar and shed light on the molecular synergy mechanisms that may have been decisive for integrating the complementary contributions of termites and their cultivar. Whether or not symbiotic and lignocellulolytic bacteria present within the comb might also contribute and complement fungal-lignin degradation capabilities is the topic of current investigations (59, 60) that will further elaborate on the question why the *Termitomyces*-termite symbiosis has become the most successful path for the termite cultivar.

## MATERIALS AND METHODS

### Genome sequencing and processing.

DNA was extracted from laboratory-grown heterocaryotic *Termitomyces* strains T112 and T153, and genome sequences were produced at LGC Genomics (Berlin, Germany) using the Illumina MiSeq V3 platform with 300-bp paired-end reads and approximately 12 million read pairs per sequencing. All library groups were demultiplexed using the Illumina bcl2fastq 2.17.1.14 software (RAW folder, Group subfolders). Up to two mismatches, or N’s, were allowed in the barcode read when the barcode distances between all libraries on the lane allowed for it. Sequencing adapters were clipped from all raw reads, and reads with final lengths of <20 bases were discarded. Afterwards, reads were quality trimmed by removing reads containing more than one N, deleting reads with sequencing errors, trimming reads at the 3′ end to get a minimum average Phred quality score of 10 over a window of 10 bases, and discarding reads with final lengths of less than 20 bp. From the final set of reads, FastQC reports were created for all FASTQ files. SPAdes version 3.13.0 was used for assembly. Prior to annotation, the genomes were soft masked with RepeatMasker 4.0.9 (61). RNA-seq data were mapped to the genomes with STAR 2.7.3a (62) and used to train the AUGUSTUS gene predictor with BRAKER 2.1.5 (63). Finally, the genomes of T112 and T153 were annotated with AUGUSTUS 3.3.3 (64). Protein and mRNA hints were used for the annotation. For details, see https://doi.org/10.5281/zenodo.4431413.

### RNA sequencing.

RNA was obtained from mycelia of *Termitomyces* strains T153 and T112 cultivated on different growth media for 10 days at room temperature. Mycelium was harvested by scraping it from agar plates with a scalpel, freezing it in liquid nitrogen, and storing it at −80°C until RNA extraction. RNA extracts underwent 100-bp paired-end BGISeq-500 sequencing at BGI (Hong Kong). For details, see https://doi.org/10.5281/zenodo.4431413.

### RNA-seq data acquisition and processing.

RNA-seq data for fresh comb (NCBI accession no. SRR5944783), old comb (accession no. SRR5944781), and nodules (accession no. SRR5944782) of *Termitomyces* strains from *Macrotermes* colony Mn156 were downloaded from the European Nucleotide Archive (65). The raw RNA-seq data were mapped to the annotated genes of T153 using HiSat2 with spiced alignments disabled (version 4.8.2) (66). Transcript abundance was then estimated using HTSeq-count (version 0.11.2) (67). Count data from HTSeq were imported into R using the DESeq2 package (version 1.22.2) (67). Genes with low transcript abundance (<10) were filtered out, and the remaining genes were log_10_ transformed (68). A heatmap for the identified redox enzymes was generated using the pheatmap package (version 1.0.12) (69) in R (70) with color schemes generated by viridis (version 0.5.1) (71). For details, see https://doi.org/10.5281/zenodo.4431413.

### CAZy analysis.

Identification of CAZymes in the predicted proteomes of *Termitomyces* and other Basidiomycetes strains was performed using a local installation of the dbCAN2 server and all three included tools (HMMER, DIAMOND, and Hotpep searches against the databases included in dbCAN2) (72). For a reliable analysis, we kept only matches that were independently identified by at least two of three annotation strategies and only genes and transcripts classified by their substrate target and thus putative enzymatic functions. EC numbers were assigned using peptide-based functional annotation (http://www.cazy.org/). For details, see https://doi.org/10.5281/zenodo.4431413.

### GC-MS analysis.

The fungal isolates *Termitomyces* sp. P5 and T153 were cultivated on solid media containing different carbon sources. GC-MS analyses of biosamples were carried out with an Agilent (Santa Clara, CA, USA) HP 7890B gas chromatograph fitted with an HP5-MS silica capillary column (30 m, 0.25-mm internal diameter, 0.50-μm film) connected to an HP 5977A inert-mass detector. For details, see https://doi.org/10.5281/zenodo.4431413.

### Activity studies of *Termitomyces* sp. T153.

Detection and quantification of H_2_O_2_ in the culture medium of *Termitomyces* sp. T153 was performed using a fluorimetric hydrogen peroxide assay kit (Sigma-Aldrich). For details, see https://doi.org/10.5281/zenodo.4431413.

### Detection of hydroxyl radicals.

Concentrations of hydroxyl radicals were measured using a fluorometric assay based on the reaction with terephthalic acid (TPA), yielding the fluorescent oxidation product hydroxy-terephthalic acid (hTPA) (for details, see https://doi.org/10.5281/zenodo.4431413).

### Ferrozine assay.

Fe^2+^ concentrations were evaluated using a standardized ferrozine assay. For details, see https://doi.org/10.5281/zenodo.4431413.

### Proteomic analysis.

*Termitomyces* sp. T153 was cultured in potato-dextrose broth (25 ml) for 12 days (20°C, 150 rpm), and secreted enzymes were collected and digested according to a standardized protocol (for details, see https://doi.org/10.5281/zenodo.4431413). LC-MS/MS analysis was performed on an UltiMate 3000 RSLCnano system connected to a Q Exactive Plus mass spectrometer (both from Thermo Fisher Scientific, Waltham, MA, USA). Tandem mass spectra were searched against the UniProt database record for *Termitomyces* sp. J132 (https://www.uniprot.org/proteomes/UP000053712; 26 November 2019) using Proteome Discoverer (PD) 2.4 (Thermo) and the algorithms of Mascot 2.4, Sequest HT (version PD2.2) and MS Amanda 2.0. The mass spectrometry proteomics data have been deposited to the ProteomeXchange Consortium via the PRIDE (73) partner repository with the dataset identifier PXD025936. For details, see 10.5281/zenodo.4431413.

### Protein analysis and activity tests.

Proteomic analysis and experimental details of laccase and MnP activity tests are deposited at https://doi.org/10.5281/zenodo.4431413.

Supporting information can be accessed free of charge at Zenodo (https://doi.org/10.5281/zenodo.4781753) and figshare (https://doi.org/10.6084/m9.figshare.14073491) and contains information regarding culture conditions, isolation procedures, structure elucidation, activity assays, expression-level data, CAZy counts, and a proteomic hit list.
